# Toward genetic modification of plant-parasitic nematodes: delivery of macromolecules to adults and expression of exogenous mRNA in second stage juveniles

**DOI:** 10.1093/g3journal/jkaa058

**Published:** 2021-02-15

**Authors:** Olaf Kranse, Helen Beasley, Sally Adams, Andre Pires-daSilva, Christopher Bell, Catherine J Lilley, Peter E Urwin, David Bird, Eric Miska, Geert Smant, Godelieve Gheysen, John Jones, Mark Viney, Pierre Abad, Thomas R Maier, Thomas J Baum, Shahid Siddique, Valerie Williamson, Alper Akay, Sebastian Eves-van den Akker

**Affiliations:** 1 Department of Plant Sciences, University of Cambridge, Cambridge, CB2 3EA, UK; 2 School of Life Sciences, University of Warwick, Coventry, CV4 7AL, UK; 3 Centre for Plant Sciences, School of Biology, University of Leeds, Leeds, LS2 9JT, UK; 4 Entomology and Plant Pathology, NC State University, Raleigh, NC 27695-7613, USA; 5 Wellcome Trust/Cancer Research UK Gurdon Institute and Department of Genetics, University of Cambridge, Cambridge CB2 1QN, UK; 6 Laboratory of Nematology, Department of Plant Sciences, Wageningen University & Research, 6708 PB Wageningen, The Netherlands; 7 Department of Biotechnology, Faculty of Bioscience Engineering, Ghent University, 9000 Ghent, Belgium; 8 Cell & Molecular Sciences Department, The James Hutton Institute, Dundee, DD2 5DA, UK; 9 School of Biology, Biomedical Sciences Research Complex, University of St Andrews, North Haugh, St Andrews KY16 9ST, UK; 10 Department of Evolution, Ecology and Behaviour, University of Liverpool, Liverpool L69 7ZB, UK; 11 INRAE, Université Côte d’Azur, CNRS, ISA, F-06903 Sophia Antipolis, France; 12 Department of Plant Pathology and Microbiology, Iowa State University, Ames, IA 50011, USA; 13 Department of Entomology and Nematology, University of California, Davis, Davis, CA 95616, USA; 14 Department of Plant Pathology, University of California, Davis, Davis, CA 95616, USA; 15 Biomedical Research Centre, School of Biological Sciences, University of East Anglia, Norwich, NR4 7TJ, UK

**Keywords:** plant-parasitic nematodes, transient expression, genetic modification, lipofection, transformation, germline

## Abstract

Plant-parasitic nematodes are a continuing threat to food security, causing an estimated 100 billion USD in crop losses each year. The most problematic are the obligate sedentary endoparasites (primarily root knot nematodes and cyst nematodes). Progress in understanding their biology is held back by a lack of tools for functional genetics: forward genetics is largely restricted to studies of natural variation in populations and reverse genetics is entirely reliant on RNA interference. There is an expectation that the development of functional genetic tools would accelerate the progress of research on plant-parasitic nematodes, and hence the development of novel control solutions. Here, we develop some of the foundational biology required to deliver a functional genetic tool kit in plant-parasitic nematodes. We characterize the gonads of male *Heterodera schachtii* and *Meloidogyne hapla* in the context of spermatogenesis. We test and optimize various methods for the delivery, expression, and/or detection of exogenous nucleic acids in plant-parasitic nematodes. We demonstrate that delivery of macromolecules to cyst and root knot nematode male germlines is difficult, but possible. Similarly, we demonstrate the delivery of oligonucleotides to root knot nematode gametes. Finally, we develop a transient expression system in plant-parasitic nematodes by demonstrating the delivery and expression of exogenous mRNA encoding various reporter genes throughout the body of *H. schachtii* juveniles using lipofectamine-based transfection. We anticipate these developments to be independently useful, will expedite the development of genetic modification tools for plant-parasitic nematodes, and ultimately catalyze research on a group of nematodes that threaten global food security.

## Introduction

Plant-parasitic nematodes are a continuing threat to food security, causing an estimated 100 billion USD in crop losses each year (Nicol *et al.* 2011). There are several different plant-parasitic lifestyles across the phylum Nematoda, the most problematic of which are the obligate sedentary endoparasites (primarily root-knot nematodes and cyst nematodes). Consequently, they are some of the most intensely studied (Jones *et al.* 2013). A current focus of the research community is to advance our understanding of plant-parasitic nematode biology in sufficient detail to develop novel methods for control. Progress in this aim is held back by a lack of functional genetic tools: forward genetics in the sedentary endoparasites is restricted to the root-knot nematode *Meloidogyne hapla*, and relies on natural variants as the source of mappable polymorphisms (Thomas and Williamson 2013) and reverse genetics is entirely reliant on RNA interference (Bakhetia *et al.* 2005), and is limited by the variable penetrance and stability of the effect. Despite these restrictions, meaningful progress recently has been made. There is nevertheless an expectation that the development of functional genetic tools would accelerate progress in understanding the biology of plant-parasitic nematodes, and thereby also indirectly the development of novel control solutions.

There are two major constraints to realizing functional genetic tools in the sedentary endoparasitic nematodes: (1) lack of knowledge and (2) biology. Firstly, the development of many functional genetic tools has been in model organisms, and thus builds on a considerable foundation of knowledge that is not yet available for plant-parasitic nematodes. For example, and to the best of our knowledge, no characterized genetic modifications have been identified in plant-parasitic nematodes that give rise to a readily scorable phenotype *in nematoda* (apart from the inability to complete the lifecycle). Secondly, the biology of plant-parasitic obligate sedentary endoparasites is generally not conducive to the technical steps required for genetic modification. Specifically, second stage juvenile (J2) hatch from eggs in the soil. At this stage, the germline in cyst nematodes consists of two non-differentiated germ cell primordia enclosed in two epithelial cap cells, and located approximately 65% of the body length from the anterior end (Subbotin and Mundo-Ocampo 2010). J2s infect the roots of plants, and cause plant tissue to redifferentiate into a nematode-induced feeding site from which nematodes withdraw all their nutrition. After induction of the feeding site, nematodes become sedentary. Sexual identity is environmentally determined: J2s that induce fully functional feeding sites at an appropriate place to connect to the vascular tissues in the roots become female, while J2s that induce smaller feeding sites, in less favorable locations, become male (Trudgill 1967). In females, the germ-cell primordia develops into a didelphic gonad. However, females become opaque, remain attached to the root for their entire life, and their germline is therefore inaccessible. In those juveniles that develop into males, a single gonad branch develops, and the animal regains motility and leaves the root in order to locate and inseminate the sedentary female nematodes. In the case of the sexual (obligate or facultative) sedentary endoparasites, males are therefore the only life stage with a mature germ line that is accessible to manipulation. Their use in hundreds of controlled crosses (Guo *et al.* 2017) confirms they are fully competent to mate. For obligate parthenogenetic sedentary endoparasites (*e.g.*, the root-knot nematode *Meloidogyne incognita*), males are produced but they do not contribute to the gene pool and, therefore, there is no life stage with a mature germline that is accessible to manipulation. Notwithstanding these challenges, the life cycle of the sedentary endoparasites is at best several weeks (*e.g., M. hapla*) and at worst several months (*e.g.*, some *Globodera* have a dormancy period between generations).

Functional genetic tools, such as CRISPR-Cas9-mediated genome editing have been developed in a number of nematode species. These include the widely studied *C. elegans* (Friedland *et al.* 2013; Frøkjær-Jensen 2013), *Caenorhabditis remanei* (Yin *et al.* 2018), and *Pristionchus pacificus* (Witte *et al.* 2015), but also relatively recently in other more challenging (animal) parasitic species (*e.g., Strongyloides* spp., *Auanema freiburgensis*, and *Auanema rhodensis*; Gang *et al.* 2017; Adams *et al.* 2019; Lok 2019; O’Halloran 2019). In this article, we aim to develop some of the foundational biology required to deliver a functional reverse genetics “toolkit” to plant-parasitic nematology. We characterized the germlines of male *Heterodera schachtii* and *M. hapla*. We tested and optimized various methods for the delivery, expression, and/or detection of exogenous nucleic acids in plant-parasitic nematodes. We demonstrate that delivery of macromolecules to cyst and root-knot nematode male germlines is difficult, but possible. Similarly, we are able to deliver oligonucleotides to root-knot nematode germlines. Finally, we show the delivery and expression of exogenous mRNA encoding various reporter genes throughout the body of *H. schachtii* juveniles using lipofectamine-based transfection. Taken together, we anticipate these developments to be useful in their own right, expedite the development of genetic modification protocol/s for sedentary endoparasitic nematodes, and ultimately catalyze research on a group of nematodes that threaten global food security.

## Materials and methods

### Biological material

Sand containing cysts of *Heterodera schachtii* was obtained from the Institute of Sugar Beet Research (IRS) in the Netherlands. Cysts were extracted from sand (by washing over 500 and 250-micron sieves with water) and collected in 50 ml falcon tubes. Juveniles were hatched by addition of 3 mM zinc chloride and incubation at 20°C in the dark. Juveniles were collected at 2–3 day intervals, and stored in 0.01% v/v Tween 20 in water at 4°C for up to 3 weeks until use.

To collect virgin males, sterile cysts were obtained from the University of Bonn (Germany), and maintained in sterile tissue culture on *Sinapis alba* (cv albatross) roots growing on KNOPs media at 20°C in the dark. Infected *Sinapis alba* roots were observed under a binocular microscope, and segments of root with differentiated J4s pre-emergence were collected, and placed in a 96-well plate. Each day, adult males that had emerged were removed and measured (for 0–1 dpe), or stored in the absence of females for *n* days until measured (for *n* days post emergence).

*Solanum lycopersicum* (cv Ailsa craig) roots infected with *M. hapla* were obtained from the University of Leeds (UK) and incubated in water in a petri dish to release males. Male nematodes were individually picked and stored in water at 4°C in the dark for up to 2 weeks until use.

### Microinjection of males

Nematodes were kept in ddH2O and washed with M9 buffer (www.wormbook.org) prior to injection. Animals were immobilized on 2–5% w/v agarose pads which were prepared by placing a drop of hot agarose on a cover slip (22 × 50 mm) and rapidly placing another cover slip diagonally on top. Once the agarose had solidified, the coverslips on top were removed and agarose pads were dried at room temperature overnight. For immobilization, a drop of Halocarbon oil 700 (Sigma H8898) was placed over the dried agar and washed worms were picked and placed in the oil using an eyelash pick. Microinjection was done using an Eppendorf Injectman and Femtojet setup on an Olympus inverted microscope with DIC prism. Worms were injected with either the Eppendorf pre-pulled Femtotips II or self-pulled needles prepared from glass capillaries (Harvard Apparatus GC120F-15) using Sutter P-2000 instrument (Settings were Heat = 290, FIL = 4, VEL = 55, DEL = 225, PUL = 110). Injections were done using the highest pressure setting on the Femtojet (clean the needle function). Hoechst stain was used at 20 mM. Cy5.5 and FITC labeled oligos were injected at 100 µM. For lipofectamine injections 8 µl of 100 µM FITC oligo, 1.3 µl of RNAIMAX reagent and 0.7 µl H_2_O were mixed. *M. hapla* cuticle is very rigid and the needle angle had to be adjusted carefully to a near perpendicular angle to the animal body axis in order to prevent the needle tip from breaking.

### Microscopy

DIC images were taken using a Leica DMI6000 B inverted microscope equipped with a DIC prism. Hoechst, Cy5.5, and FITC images were taken using either a Leica SP5 or SP6 confocal imaging system. Live animals were placed on 2% w/v fresh agarose pads with a 5 µl drop of 10 mM Levamisole and covered by a coverslip.

### Delivery of mRNA to plant-parasitic nematodes by lipofection

Capped and polyadenylated mRNAs encoding eGFP or Firefly luciferase were obtained from Ozbiosciences (codon table, UTR sequence, and ribosomal binding site are not disclosed by the supplier). To aid transfection of reporter mRNAs, three lipofection agents were individually used according to the manufacturer’s instructions. Approximately 15,000–20,000 J2 *H. schachtii* were soaked for 24 h for eGFP and mCherry: in 500 ng of mRNA, 3% lipofectamine RNAIMAX, MessengerMAX, or CRISPRMAX (Invitrogen), 100 mM octopamine (Thermo-Fisher), in a total volume of 50 µl (adjusted with Opti-MEM^™^ I Reduced Serum Medium [Invitrogen]). For luciferase assays, 16 µg of mRNA, 12% lipofectamine RNAIMAX, and 100 mM octopamine (Thermo Fisher), were used in a total volume of 30 µl (adjusted with nuclease free M9 buffer).

### Detection of eGFP

Live transfected nematodes were transferred to a 76 × 26 mm microscope slide (Thermo scientific). The expression of eGFP was measured using a Leica SP5 confocal system mounted on a DM6 microscope equipped with an argon laser and photomultiplier tube (PMT) detectors. The negative control in the fluorescence assay substituted the mRNA encoding a fluorescent protein at 476 nm (eGFP) with an equal quantity of mRNA encoding a non-fluorescent protein at 476 nm (mCherry). Z-stack images of the nematodes were collected with a 5 µm interval (ex 476 nm, em 508–513 nm, gain 714). Fluorescence difference between treated and control nematodes was visualized qualitatively. To provide a quantitative measure of fluorescence, the most in-focus optical section from the Z-stack was selected for each nematode manually. The image of each nematode was cropped out, inverted, their brightness, contrast, and intensity adjusted for both treatment and control (Brightness −17%, Contrast + 71%, and Intensity −27%), and the number of pixels per nematode that exceeded an empirically derived number of shades of variance (an integer between 0 and 255) from the background gray (Hexadecimal color code 0 × D1D1D1) were counted using the PixelSearch function in a custom AutoIT script (https://github.com/sebastianevda/ColourCounter). The numerical value assigned to each nematode’s fluorescence allowed us to test the significance of the difference between the treatment and the control using an independent two-group Mann–Whitney *U* test.

### Detection of luciferase expression

Luciferase expression was detected using the CLARIOstar plate reader (gain 4095). The supernatant was removed from the soaked nematodes. Each set of animals was resuspended in 240 µl nuclease-free M9 buffer, and distributed over 16 wells; eight positive (mRNA soaked) and eight negative controls (no mRNA). Using a CAPP^®^ 8-Channel Pipette (Starlab) 10 µl of 100 mM VivoGlo Luciferin (Promega) was added to the negatives and then to the mRNA soaked worms. The CLARIOstar plate reader was set up to vortex after each measurement at 300 rpm, and the plate was sealed with Corning^®^ microplate sealing tape (Sigma) and loaded into the machine and measured every 176 s. To quantitatively compare the dependency of luminescence as a function of time, the following model was fitted to the 16 time series (8 series for mRNA soaked, 8 for controls). The formula: Intensity = a + b * 2^(c * time). The model was fitted by the following R command: nls(y ∼ a + b * 2^(c * time), start = list(*a* = 1000, *b* = 1000, *c* = −0.0001)). The obtained half-lives (−1/c) in mRNA soaked is compared with the control half-lives using the independent two-group Mann–Whitney *U* test.

### Data availability

NGS reads deposited under ENA accession PRJEB39266. Scripts available at github repositories https://github.com/sebastianevda/ColourCounter and https://github.com/OlafKranse/Selective-analyses-of-areas-of-interest-for-next-generation-sequencing. Gene sequences available at DRYAD under accession doi: 10.5061/dryad.r4xgxd296.

Supplementary material id available at figshare DOI: https://doi.org/10.25387/g3.13186817.

## Results

### Characterization of the gonad of adult motile plant-parasitic nematodes

In order to develop a procedure for the genetic modification of plant-parasitic nematodes, we need to deliver macromolecules to the germline. Given that: (1) females of sedentary obligate biotrophs (including root-knot and cyst nematodes) are opaque and inaccessible (Supplementary File S1) and (2) males are transparent, regain motility to mate with females, and are in principle accessible for manipulation, we characterized the germlines of male *Heterodera schachtii* and *M. hapla* to guide the delivery of macromolecules.

**Figure 1:**
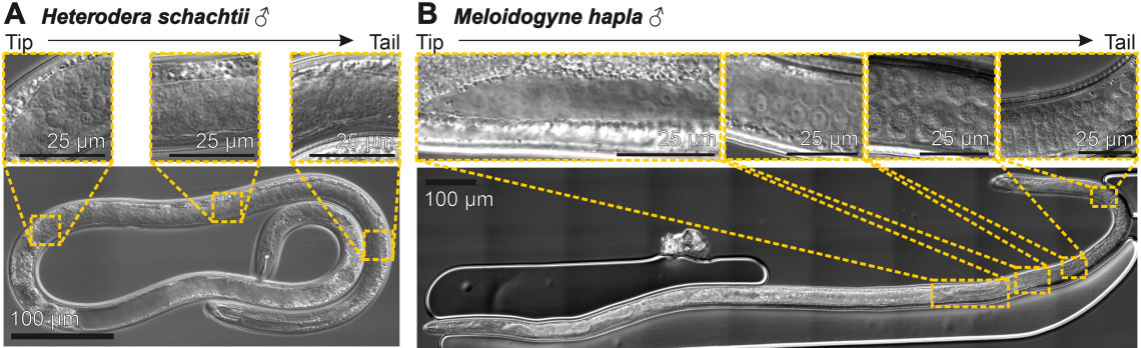
The gonad of cyst and root-knot nematode males. (A) *H. schachtii* male. Uniformity of germ cell size and shape from distal tip to proximal end is shown in three inset panels. (B) *M. hapla* male. Variation in germ cell size and shape from distal tip to proximal end is shown in four inset panels.

Generally, the gonad of *H. schachtii* and *M. hapla* males is single ended, occupies most of the volume of two thirds to one-third of the nematode body length, and appears cellularized, rather than syncytial, throughout. Notwithstanding the considerable variation within species, *M. hapla* males are approximately twice as long and twice as wide as *H. schachtii* males. Most notably, the morphology of germ cells varies considerably between *H. schachtii* and *M. hapla*. The morphology of *H. schachtii* germ cells is uniform from distal tip to proximal end of the germline (Figure 1A). This is consistent with the completion of meiosis prior to the final molt to adult male (Kempton *et al.* 1973). Viewed under DIC-microscopy, the *H. schachtii* germ cells appear as irregular polygons with compact nuclei reminiscent of spermatids in *C. elegans*, and are tightly packed along the entire gonad. Up to four can be found abreast within the gonad of *H. schachtii.* In contrast, germ cells of *M. hapla* vary considerably from the distal tip to the proximal end of the gonad (Figure 1B) as continued sperm development can occur after adult males regain motility (Shepherd and Clark 1983). At the distal tip of the *M. hapla* gonad, individual spherical cells are evident, less tightly packed than in *H. schachtii*, and surrounded by a matrix. In the mid-gonad, the cells are larger than at the distal tip and assume an irregular pentagonal shape when viewed under a DIC microscope. More posteriorly, the cells are larger still (only two can be found abreast within the much wider germline of *M. hapla*) and the contents appear granular. These cells resemble spermatocytes of *C. elegans*. Toward the proximal end of the *M. hapla* gonad, the cells are not unlike those found throughout the *H. schachtii* germline: they appear irregular polygons, the nuclei are compact, and they are packed approximately 3–4 abreast in the gonad (therefore approximately twice the size of those in *H. schachtii*). These cells resemble spermatids of *C. elegans* (L’Hernault 2006). Comparison between the two species indicates the presence, and location, of cells at different stages of development in the *M. hapla* emerged male gonad, which are absent in *H. schachtii* emerged male gonad. These data guide the general location for the delivery of macro molecules (toward the distal tip), and may result in differences in accessibility of cells within the germline to genetic modification.

**Figure 2:**
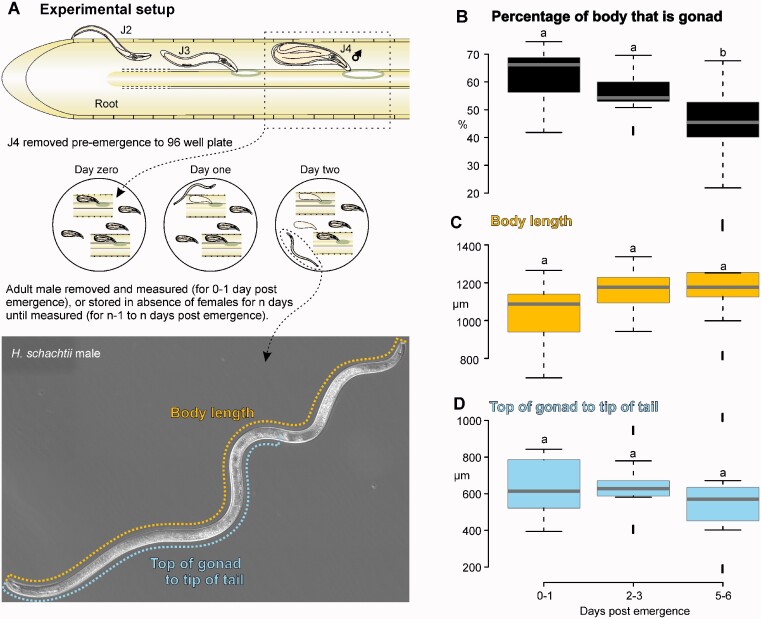
Measuring the change in *H. schachtii* male gonad over time. (A) Schematic representation of experimental setup to collect differentiated males pre-emergence with representative measurements of *H. schachtii* males and germline. (B–D) Comparison of germline and body length at 0–1, 2–3, and 5–6 days postemergence of virgin males showing percentage of body length occupied by germline, body length, and gonad length, respectively. Horizontal line in box plot represents median value, whiskers extend to data points that are less than 1.5 × IQR away from 1st/3rd quartile. Different lowercase letters indicate significant differences at *P* < 0.05 (Mann–Whitney *U* test).

Initial observations revealed considerable variation in the proportion of the *H. schachtii* male body occupied by the germline. To characterize this phenomenon, we developed a method to collect virgin males at specific times postemergence (Figure 2A). In brief, segments of roots with differentiated males that had not yet emerged, or those dislodged from the root, were collected and stored in PBS at room temperature in a 96-well plate. Adult males that had emerged were removed each day from the 96-well plate, and stored in the absence of females until measured. Males were imaged under DIC microscopy, and measured using FIJI software (*e.g.*, Figure 2A). Notwithstanding large overall variation in absolute body size, the proportion of the body that is occupied by the germline decreases over time from approximately 65%, to approximately 45% (Figure 2B, *n* = 7–9, Mann–Whitney *U*, *P* < 0.05). The decrease in the proportionate length of the gonad is likely a combination of more modest but individually not significant increases in body length and decreases in gonad length (Figure 2, C and D).

As the gonad becomes proportionally shorter, we observed the progressive appearance of large vacuous structures between the head and the distal tip of the gonad in virgin males, with this starting almost immediately after emergence (Supplementary Figure S1). Male nematodes (presumably some nonvirgin) with large vacuous structures can be recovered from infected roots, and so we reason this is not a function of the buffer they were stored in prior to imaging, but rather a natural phenomenon.

### Delivery of macromolecules to male gonads

**Figure 3:**
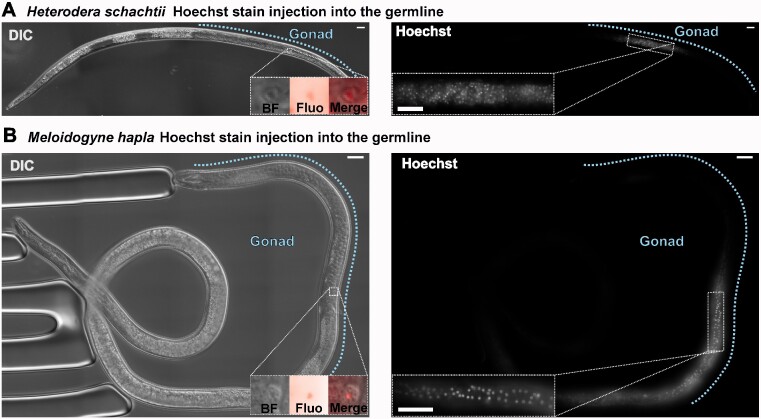
Microinjection of fluorescent dye to adult male gonad of *H. schachtii* and *M. hapla*. Brightfield left, fluorescence right. Gonads indicated by blue dotted lines. Inset in the left panel shows a digital zoom of a single cell within the gonad (brightfield [BF], fluorescence [Fluo], and overlay [Merge]). Inset in the right panel shows a digital zoom of a region of the gonad. (A) *H. schachtii* male. (B) *M. hapla* male. Scale bars indicate 20 μm.

Based on our characterization of the gonads of *H. schachtii* and *M. hapla* males, young individuals were selected from pools of mixed age emerged males for injection. After considerable optimization of injection conditions (detailed in Supplementary File S1), we were successful in delivering a membrane permeable DNA dye (Hoechst) to both *H. schachtii* and *M. hapla* male gonad (Figure 3). In both cases, the dye remains local to the site of injection, is taken up by cells, and stains the nuclei. The observed number and size of fluorescent foci is characteristic of each species’ germline (*H. schachtii* small and numerous, *M. hapla* fewer, larger cells with less dense nuclei).

**Figure 4:**
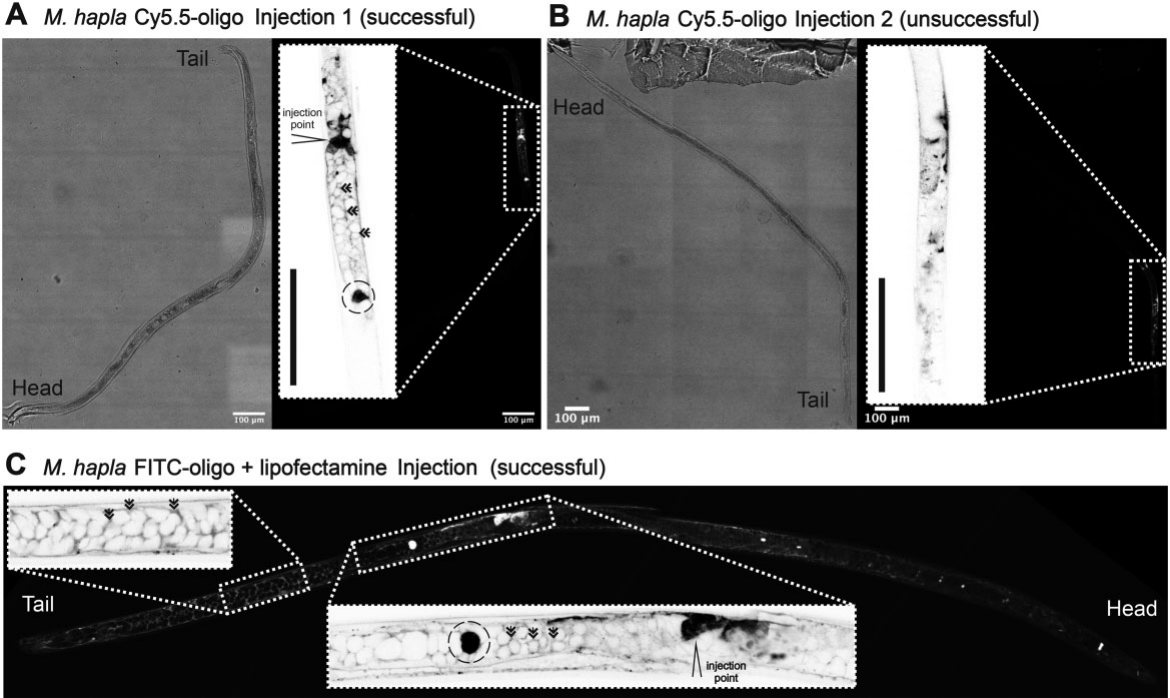
Microinjection of fluorescent-tagged oligonucleotides to the *M. hapla* male germline. (A) and (B) successful and unsuccessful injection of oligonucleotides-tagged with Cy5.5. Brightfield left, fluorescence right (inset inverted high magnification image of injection site marked for successful injection). (C) Fluorescence image of successful injection of oligonucleotides tagged with FITC [inset inverted high magnification image of germline tail end (left) and successful injection site (right, marked)]. Double arrows indicate most fluorescent-tagged oligonucleotides collect in the space around cells. An example of extremely bright cells, perhaps indicative of uptake, are highlighted with broken circles. Scale bars indicate 100 μm.

To further explore macromolecule delivery to the more accessible *M. hapla* gonad, oligonucleotides (Supplementary Table S1, 63 nt) were synthesized with the covalent addition of 5′ Cy5.5 (excitation 675 nm and emission 694 nm) or FITC (excitation 495 nm and emission 519 nm). Examples of apparently successful and unsuccessful injection of Cy5.5-tagged oligo to the *M. hapla* germline are shown in Figure 4, A and B, respectively (determined by visible expansion of the worm following injection and detection of abundant fluorescence in the injection site). Similar to injection of Hoechst, the material remains local to the site of injection. In a successful injection, the majority of the oligo remains in the space between cells in the germline, and highlights their characteristic shape (*cf.*Figure 1B). For most successful injections, one, or very few, individual cells proximal to the injection site were extremely bright, perhaps indicative of uptake (Figure 4A).

We explored the use of liposomes to facilitate delivery of macromolecules. Liposomes are vesicular lipid bilayers that have long been used to deliver various cargo to cells by transfection (Felgner *et al.* 1987), and more recently to aid microinjection (Adams *et al.* 2019). We therefore encapsulated FITC-oligo into liposomes (RNAiMAX lipofectamine) before injection, and injected into the gonad of male *M. hapla* (Figure 4C). Liposomes are vesicular lipid bilayers that have long been used to deliver various cargo to cells by transfection (Felgner *et al.* 1987), and more recently to aid microinjection (Adams *et al.* 2019). The resulting fluorescence does not remain local to the site of injection, unlike the injection of the Cy5.5 oligo. However, similar to injection of the Cy5.5 oligo, the majority of the fluorescence remains in the space between cells in the germline, and highlights their characteristic shape. Occasionally, individual cells within the germline, proximal to the injection site, are extremely bright, perhaps similarly indicative of uptake (Figure 4C). This is different in appearance to the injection of Hoechst (Figure 3) because Hoechst is a cell permeable dye that specifically stains nuclei (minimal fluorescence in the cytoplasm but becomes highly fluorescent when bound to DNA), while the oligos are fluorescent in solution and therefore also in the cytoplasm.

### Lipofection-based delivery and expression of mRNA in *H. schachtii* second stage juveniles

Given that males are extremely challenging to inject, in particular for *H. schachtii*, we explored various other methods to deliver macromolecules to plant-parasitic nematodes (Supplementary File S1). The most promising approach was a lipofection-based method to deliver macromolecules to J2s. mRNAs encoding reporter proteins were selected based on two factors. (1) the availability of commercially available mRNAs commonly used in transfection assays and (2) rapid and extremely sensitive detection of expression of the encoded protein in live animals using photomultiplier tubes (*e.g.*, confocal microscopy and bioluminescence plate reader). mRNAs encoding enhanced GFP (eGFP) were packaged into liposomes and delivered to *H. schachtii* J2 by *in vitro* soaking for 24 h. Soaked nematodes were washed and imaged using confocal microscopy. On an inverted grayscale, nematodes soaked with mRNA encoding eGFP encapsulated in liposomes were qualitatively darker (*i.e.*, have more fluorescence) than those soaked in lipofectamine alone. However, fluorescence exists in all imaged nematodes (autofluorescence), particularly in the intestine. A quantitative approach was developed to differentiate between eGFP fluorescence and autofluorescence. In brief, grayscale fluorescence images of individual nematodes were extracted, inverted, and the contrast of all cropped images adjusted. Pixels exceeding an empirically derived brightness threshold (see *Materials and Methods* for details) were counted and marked (see *Materials and Methods* and Supplementary Figure S2).

**Figure 5:**
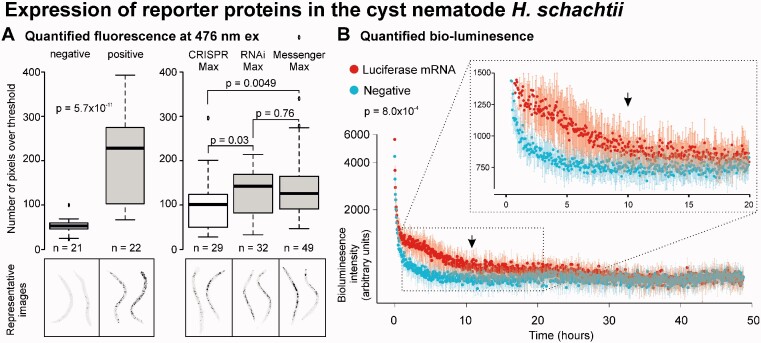
Expression of exogenous mRNAs in second stage juvenile *H. schachtii*. (A) Quantification of fluorescence. Representative images of negative control and treated nematodes. Pixels above threshold marked (black) and counts are shown in the boxplot. Horizontal line in box plot represents median value, whiskers extend to data points that are less than 1.5 × IQR away from 1st/3rd quartile. Left, nematodes fixed post lipofection comparing nematodes soaked in empty liposomes (negative) with nematodes soaked in liposomes containing mRNA encoding GFP. Right, live nematodes comparing mRNA encoding GFP encapsulated in either CRISPRMAX or RNAiMAX lipofectamine. *P*-values are indicated for independent two-group Mann–Whitney *U* test. (B) Quantified bio-luminescence (arbitrary units plotted on log scale) of live nematodes soaked in mRNA encoding luciferase encapsulated in liposomes (red), or nematodes soaked in empty liposomes (turquoise), measured every 176 s for 48.84 h. Inset, a zoom in of h 0–20. Arrow indicated 10 h. The half-life in treated nematodes is compared with the control using the independent two-group Mann–Whitney *U* test. Error bars indicate standard deviation of the mean (*n* = 8) at each time point.

Using this method, the intensity of fluorescence between mRNA soaked worms and control soaked worms was quantified and compared. From the representative images in Figure 5A, a clear difference was observed in the fluorescence intensity between the control and mRNA soaked worms. Nematodes soaked with lipofectamine MessengerMAX containing mRNA encoding a fluorescent protein excited at 476 nm (eGFP) were on average 3.9 times brighter than nematodes soaked in lipofectamine containing mRNA encoding a non-fluorescent protein at 476 nm (*n* = 21 and 22, respectively, Mann–Whitney *U P* = 5.703e−11). From the marked pixels, we can see that: (1) Most autofluorescence in control nematodes is, as expected, restricted to the digestive system of the nematode and (2) the observed increase in fluorescence in treated nematodes is not just in the digestive system of the nematode, but also throughout the body. In an independent experiment, a comparison was made between the efficacy of three different types of lipofectamine, MessengerMAX, RNAIMAX, and CRISPRMAX (Figure 5A). Both MessengerMAX (*n* = 49, Mann–Whitney *U P* = 0.004996) and RNAIMAX (*n* = 32, Mann–Whitney *U P* = 0.03084) outperformed CRISPRMAX (*n* = 30) in overall fluorescence (Figure 5).

To determine the potential lifetime of transient expression, we repeated the experiment with mRNA encoding luciferase encapsulated in RNAIMAX liposomes and measured nematode light emission every 176 s for 2 days using a CLARIOstar plate reader. Nematodes continued to emit light above background noise luminescence for at least 10 h, and possibly up to 30 h (Figure 5B), beyond which the treated and control nematodes were indistinguishable, presumably as substrate, mRNA, or both, are consumed. The decrease in luminescence as a function of time of treated and control nematodes was compared using a Mann–Whitney *U* test for unpaired data (*P* = 8.0*10^−4^).

## Discussion

Obligate sedentary endoparasites are the major contributor to the worldwide crop losses caused by plant-parasitic nematodes, and so are intensely studied. Developing a system to genetically transform them should therefore be top priority, but their biology makes them one of the most difficult species in which to achieve this.

The biology and gonad accessibility are two major barriers to transformation of sedentary endoparasitic plant parasites: the germlines are either not developed enough (juvenile), inaccessible (female), or apparently non-syncytial when accessible (males). Most successful examples of using microinjection to transform nematodes are on species with accessible syncytial gonads (*e.g., Caenorhabditis* spp., *Pristionchus* spp., and the animal parasite *Strongyloides* spp.). One exception may be *Auanema s*pp., although it is not entirely clear whether the gonad is syncytial or not (Adams *et al.* 2019). In *H. schachtii* and *M. hapla*, males are the only accessible life stage with a developed germ line (albeit non-syncytial). In general, male root-knot and cyst nematodes are technically hard to inject: their cuticles are hard, their body is non-elastic, and they appear to be under high internal pressure so that delivering contents from the needle is difficult and unreliable. *Heterodera schachtii* males are less suitable for microinjection than *M. hapla* males, because the former are smaller and their germline cells are fully differentiated, with all cell divisions occurring before the final molt and only spermatids and spermatozoa present in the adult gonad (Kempton *et al.* 1973) and they are less able to accommodate injections. In contrast, all stages of sperm development are present in the adult male gonad of *M. hapla*, from spermatogonia at the tip to mature spermatozoa at the proximal end (Shepherd and Clark 1983). Nevertheless, we have demonstrated the delivery of a membrane-permeable fluorescent dye to *H. schachtii* and *M. hapla* male gonad, and the delivery of oligonucleotides to *M. hapla* male gonads. It should be emphasized that while this is possible, it is not routine, and most cells in the male *M. hapla* gonad do not spontaneously uptake even these short oligonucleotides. Recent demonstrations in animal parasitic nematodes have shown that the inclusion of lipofectamine in the injection mix, and subsequent injection into the gonad of *Strongyloides stercoralis* enabled CRISPR-Cas9 genome editing throughout the body (Adams *et al.* 2019). Our results suggest that including lipofectamine in the injection mix with fluorescently tagged oligonucleotides may increase the intercellular spread of the fluorescence, but we find no clear increase in cellular uptake in these species and at the concentrations used. It is possible that most cells in the gonad are too far differentiated to be receptive to cargo.

Despite their immature germline, the only other life stage of the sedentary endoparasites that is accessible to manipulation would be the J2. We explored whether lipofectamine could be used to deliver cargo to J2s by encapsulating mRNA encoding reporter genes in liposomes and using octopamine to stimulate nematode ingestion. Using this approach, we were able to demonstrate, for the first time, expression of exogenous mRNAs encoding fluorescent or bioluminescent proteins in a plant-parasitic nematode. Both fluorescence and bioluminescence are extremely sensitive methods of detection. While clearly above the background autofluorescence/noise thresholds for each technique, there is much that can be done to improve the signal to noise ratio, and thereby the utility of the approach. We expect major improvements in efficiency by optimizing the reagents, for example, the codon usage of mRNAs for *H. schachtii*, the inclusion of *H. schachtii* UTRs, and the use of lipofectamines specifically designed to encapsulate mRNAs. It is possible that modification of the mRNA may also improve stability and translation *in vivo* (Boo and Kim 2020). We also expect there is scope for improvement by optimizing the experimental setup (*e.g.*, time-of-soaking and time-of-detection, mRNA concentration, lipofectamine concentration, etc.). Finally, we expect that addition of a nuclear localization signal (in the case of fluorescent reporters) or epitope tag/s (in the case of bioluminescent reporters) may help to concentrate the signal, *in vivo* or *in vitro*, respectively. Future optimization may also include the lipofection-based transfer of DNA, as opposed to mRNA. Delivery of DNA could have several benefits over mRNA. Protein expression from a DNA may be higher than from an mRNA because one molecule of mRNA delivered to a cell equates to at best one molecule of protein, but one molecule of DNA delivered to a cell could equate to many molecules of mRNA and therefore protein. Most importantly however, delivery of DNA may allow tissue-specific expression profiles to be achieved when combined with promoters, or elements therein (Eves-van den Akker and Birch 2016; Espada *et al.* 2018), making additional use of the abundance of available genomic resources for plant-parasitic nematodes (Abad *et al.* 2008; Opperman *et al.* 2008; Kikuchi *et al.* 2011; Cotton *et al.* 2014; Eves-van den Akker *et al.* 2016; Phillips *et al.* 2017; Mathew and Opperman 2019). However, delivery of DNA places one additional barrier between nucleic acid and protein (the nucleus), and so it is yet to be determined whether, overall, it is superior.

The ability to transiently express exogenous mRNAs in plant-parasitic nematodes is important and so worth optimizing. Not only is it technically trivial and readily adopted without any specialized equipment, but it would also enable several experimental approaches that, until now, have been either impossible or prohibitively difficult for plant-parasitic nematology: *in vivo* protein-DNA interaction studies (ChIP seq) and *in vivo* protein-protein interaction studies (Co-IP, BiFC, FRET, *etc.*). Most importantly, the observed increase in fluorescence in treated nematodes is throughout the body, not just in the digestive system of the nematode. This means that with sufficient optimization of the technique, it may be possible to achieve expression of exogenous mRNA in, for example, the germ cell primordia of juvenile worms. Delivery in this way would avoid the difficulties of injecting cargo into male gonads, the subsequent uptake into male germ cells, the unknown challenges associated with mating females with males postinjection, and in the case of the obligate parthenogens (*e.g., M. incognita*) the fact that males appear to not contribute genetically. Taken together, this transient expression system may also enable heritable genetic modification of plant-parasitic nematodes, through the delivery of mRNA encoding CRISPR-Cas variants to the germ cell primordia of juveniles. By the same token, it may be possible to achieve expression of fluorescent proteins in the esophageal gland cells, and thereby explore aspects of plant-parasitic nematode biology that have been technically intractable, for example, effector delivery, in real time.

We attempted to use a similar lipofection-based technique to deliver CRISPR-Cas9 protein to somatic cells of *H. schachtii* juveniles, but this was not successful (detailed in Supplementary File S1). We initially tried to introduce a known edit by HDR, but the frequency of PCR-derived template switching was prohibitively high. We proceeded with an experiment designed to introduce a range of unknown edits by NHEJ in somatic cells of *H. schachtii* juveniles but were unable to consistently identify edited events. There are several potential explanations for these difficulties. The edited events are likely rare: if they occur at all, they may only be in a few cells per juvenile (PCR amplification of the locus does not selectively amplify edited events). Edits in individual cells are independent, and therefore may be different (even improving editing efficiency from 1 cell to 10 cells per nematode does not necessarily increase the signal, as each edit could be different). Targeting an endogenous endonuclease restriction site for mutagenesis would allow the digestion of unedited DNA, and should increase sensitivity of detection of rare events. To the best of our knowledge, there are no known genetic modifications that would result in selectable dominant phenotypes in cyst or root knot nematode juveniles. Since the optimal temperature for *Streptococcus pyogenes* Cas9 (SpCas9) is around 37°C, we envision that a heat-shock treatment may increase its efficiency (Xiang *et al.* 2017; LeBlanc *et al.* 2018). An additional possibility is to increase the accessibility of the enzyme to the target sequence by inducing an “open” chromatin state (Chen *et al.* 2017; Park *et al.* 2020). Finally, but perhaps most importantly, a successful CRISPR experiment requires several steps: (1) delivery of components into cells, (2) targeting a gene amenable to CRISPR, including guides that work *in vivo*, and (3) detection of possibly rare events—and failure at any one stage will result in failure. None of these steps have been established for any gene in plant-parasitic nematodes. It seems prudent to isolate and optimize as many of these challenges as possible. We anticipate that an optimization of the protocol for lipofection-based delivery to juveniles described here will provide a route to rapidly overcome some of these challenges.

## Conclusions

Genetic modification of sedentary endoparasitic nematodes is an ongoing challenge. Delivery of cargo to their gonad by microinjection is difficult, but not impossible. Expressing exogenous mRNA throughout the juvenile body of *H. schachtii* is technically trivial, and potentially useful either on its own, or as a route to expedite the development of genetic modification protocol/s for sedentary endoparasitic nematodes.

## Funding

Work on plant-parasitic nematodes at the University of Cambridge is supported by DEFRA licence 125034/359149/3, and was funded by Biotechnology and Biological Sciences Research Council (BBSRC) grants BB/R011311/1, BB/N021908/1, and BB/S006397/1, a Synthego Genome Engineer grant, and a Genewiz grant. A.A. was supported by a Wellcome/Newton trust Institutional Strategic Support Fund grant and a UKRI Future Leaders Fellowship MR/S033769/1. C.J.L. was supported by BBSRC grant BB/N016866/1. J.J. receives funding from the Scottish Government Rural and Environmental Science and Analytical Services division. T.R.M. and T.J.B. were supported by grants from the Iowa Soybean Association and by State of Iowa and Hatch funds. A.P.S. and S.A. were supported by grants RPG-2016-089 and BB/L019884/1.

*Conflicts of interest*: None declared.

## Supplementary Material

jkaa058_Supplementary_Data
