# Supplementary material for: Toward genetic modification of plant-parasitic nematodes: delivery of macromolecules to adults and expression of exogenous mRNA in second stage juveniles
Source: G3 (Bethesda). 2021 Feb 15;11(2):jkaa058. doi: 10.1093/g3journal/jkaa058 (PMC8022973; doi:10.1093/g3journal/jkaa058)
Supplement: jkaa058_Supplementary_Data [file jkaa058_supplementary_data.docx]

figshare DOI: <https://doi.org/10.25387/g3.13186817>

Flybase? NO Wormbase? NO Saccharomyces Genome Database? NO FungiDB? NO.
